# Cardiac Syncope with Anoxic Seizure Activity

**DOI:** 10.1155/2020/6749382

**Published:** 2020-01-08

**Authors:** Samed Talibi, Craig Douglas, Benjamin Pope

**Affiliations:** ^1^St1 Neurosurgery, Department of Emergency Medicine, Queens Medical Centre Nottingham, Nottingham, UK; ^2^Department of Emergency Medicine, Queens Medical Centre Nottingham, Nottingham, UK

## Abstract

This is a case report, which explores the presentation to the emergency department of a fit and well female with a known ventricular bigeminy. She presented with convulsive episodes. The working differential diagnosis was of possible cardiac syncope with anoxic seizure activity or neurogenically mediated arrhythmia secondary to subarachnoid haemorrhage. On further collateral history, the patient was on citalopram. The ECGs demonstrated PVCs of multiple morphologies that were transiently bidirectional, raising the possibility of catecholaminergic polymorphic ventricular tachycardia. The presentation of a young fit patient with syncope and seizure-like episodes should always raise concern for the admitting emergency medicine clinician of an underlying cardiac pathology.

## 1. Introduction

Ventricular bigeminy is described on electrocardiography (ECG) as an alternating pattern of normal sinus rhythm and premature ventricular complexes (PVCs). PVCs are characterized by the premature occurrence of a QRS complex that is abnormal in shape and lasts greater than 120 milliseconds [1]. The T-wave that follows this is large and usually of opposite polarity to the QRS complex. Most PVCs do not affect the sinus node so there is usually a postectopic compensatory pause or the PVC may be interpolated between two sinus beats as seen in bigeminy [1].

If there are three or more successive PVCs, this is defined as nonsustained ventricular tachycardia (VT) [1, 2].

PVCs are very common, even in patients without evidence of heart disease. PVCs become more prevalent with increasing age, and they are commonly seen in patients with ischaemic heart disease (IHD) and almost universally seen in patients who have suffered myocardial infarction (AMI). PVCs also occur in a range of other settings including patients exposed to sympathomimetic drugs, patients with alkalaemia or hypoxia, and patients with congestive cardiac failure (CCF). Most patients with PVCs, bigeminy, or indeed trigeminy do not require treatment, and the most important aspect of care is to treat any underlying diagnosis.

This is a case report that explores the presentation to the emergency department of a fit and well female with known ventricular bigeminy.

## 2. Case Report

A 44-year-old female presented to the emergency department (ED) with an episode of syncope occurring on standing from a crouched position. The patient reported feeling dizzy and light headed before she collapsed, and collateral history from onlookers described a 3–5 minute episode where the patient's limbs were jerking, her eyes were rolling, and she was incontinent of urine. The length of the postictal period was unclear, but the patient was complaining of bifrontal headache.

The past medical history included asthma for which she used inhalers infrequently and psoriasis. There was a possible diagnosis of depression as she had been recently commenced on citalopram by the GP. She had never required a hospital admission for asthma but had previously seen a cardiologist because of bigeminy. She was a nonsmoker and drank small amounts of alcohol.

Her examination was largely unremarkable, of note her heart rate was 50 bpm and blood pressure 95/61 mmHg. The admission ECG showed sinus rhythm with monomorphic PVCs.

Shortly after arrival in the ED, she had a second event. She complained of feeling light headed before losing consciousness on the trolley. She was witnessed to be stertorous and required a jaw thrust to open her airway. She was hypertonic with flexed upper limbs. She was noted to be bradycardic and hypertensive during the postictal period. On recovery, she described no preceding symptoms of chest pain or palpitations.

The initial differential diagnosis was of possible cardiac syncope with anoxic seizure activity or neurogenically mediated arrhythmia secondary to subarachnoid haemorrhage. She was transferred for a CT scan of the head, during which she experienced a cardiac arrest. The patient started to show signs of life during the first cycle of chest compressions, and cardiac output was maintained; GCS steadily improved. The initial postarrest ECG showed normal sinus rhythm with multifocal PVCs; however, on continued ECG monitoring, it became clear that she was experiencing runs of nonsustained polymorphic VT. Her CT head and subsequent CT venogram did not show an acute bleed or venous sinus thrombosis but a tiny focus of high attenuation in the anterior limb of the right internal capsule that may represent calcification. There was an associated developmental venous anomaly suggesting the possibility of a small cavernoma.

Reviewing her family tree, there was no clear pattern of congenital heart disease or sudden cardiac death.

She was initially managed with IV magnesium sulphate infusion, temporary pacing to suppress ventricular arrhythmias, and discontinuation of citalopram.

On review of this admission, ECG's QTc seemed to be either towards the upper end of normal or mildly prolonged (470–504 ms); however, it was difficult to assess due to the high frequency of her PVCs. When the patient's medical records were available, previous ECGs demonstrated corrected QT intervals in the region of about 470 ms, towards the upper limit of normal for a female. It became apparent our patient had been seen by cardiology a number of years ago, for review of what at that time were felt to be monomorphic ventricular ectopics. The ECGs on this presentation, however, demonstrated PVCs of multiple morphologies that at one point looked bidirectional, raising the possibility of catecholaminergic polymorphic ventricular tachycardia.

The patient was admitted to the coronary care unit where she had coronary angiography and cardiac MRI which were both normal. She also had electrophysiology studies that ruled out the presence of an accessory pathway.

She had a dual-chamber ICD implanted and was discharged on nadolol 80 mg daily.

## 3. Discussion

PVCs are common and largely asymptomatic but can cause palpitations, presyncope, and/or syncope in rare circumstances [1, 2]. In patients with structurally normal, healthy hearts, their occurrence is usually associated with no clinical significance. The presence of frequent PVCs, however, in some patients, especially those with a background of heart disease, may suggest the potential for deterioration to a malignant arrhythmia.

The prevalence of PVCs in the general population is 1% in clinically well patients as detected by a standard 12-lead ECG. Prevalence has been recorded as high as 75% in healthy persons if a 24–48 hour ambulatory (Holter) ECG is performed [2, 3].

A source of possible confusion is the Framingham Heart Study where PVCs were associated with a twofold increase in the risk of all-cause mortality, MI, and cardiac death. However, it is important to realize the impact of confounding factors on these results, in particular the presence of ischaemic and structural heart disease within this patient cohort [2, 3].

In polymorphic VT (PMVT), the QRS complexes have many different shapes in one monitoring lead, and torsades de pointes is a specific variant of this condition in which the QRS complexes swing from a positive to negative direction. It usually occurs in short runs of 5–15 seconds in patients with severe myocardial disease which prolongs ventricular repolarization or patients with inherited or acquired prolonged repolarization, i.e., long QT syndromes. It may itself cause cardiac arrest, as may have been the case with our patient or further deteriorate to ventricular fibrillation [4–6]. PMVT or torsades being precipitated in this setting of an early depolarization coinciding with prolonged repolarization, the so-called R-on-T phenomenon is well described. However, what is much rarer and subsequently appreciated to a much smaller degree is the risk of early depolarization precipitating PMVT in the setting of normal ventricular repolarization and hence a normal QTc on the ECG. This is exactly what occurs in catecholaminergic PMVT [7–9].

Catecholaminergic PMVT is a genetic arrhythmogenic disorder with a prevalence of 1 in 10,000 [7]. Patients will present with syncopal episodes and with a distinctive pattern of highly reproducible, stress-related, bidirectional ventricular tachycardia in the absence of both structural heart disease and a prolonged QT interval. The ECG trace pattern closely resembles the arrhythmias associated with calcium overload and the delayed after-depolarizations observed during digitalis toxicity [7, 10]. Mutations in genes that handle calcium homeostasis in cardiac myocytes are disrupted, and this impairment can lead to ventricular tachycardia. It is a significant cause of sudden cardiac death in children and young adults without recognized heart abnormalities [7, 10].

Catecholaminergic PMVT is a rare diagnosis and was not considered in this case until the patient was reviewed by a consultant cardiologist. The working diagnosis until that time from the emergency clinicians was of PMVT secondary to prolonged QTc induced by citalopram even though our evidence of a prolonged QTc was fairly weak. With respect to this working diagnosis, Zeltser et al. in 2003 found that 96% of patients who had torsades de pointes associated with a noncardiac drug had at least 1 concomitant risk factor and 71% of patients had at least 2 risk factors [11]. These risk factors included: prolonged QTc interval > 500 ms, female gender, left ventricular systolic dysfunction, elderly, hypokalemia/hypomagnesemia, bradycardia, drug induced or organ dysfunction elevating plasma concentrations of QT-prolonging drugs, concomitant administration of more than 1 drug that is known to prolong QT, and genetic predisposition [12, 13]. Our patient certainly was female and for the most part was bradycardic. It could be argued that she had a QTc more than 500 ms, but this was an inconsistent finding. Despite magnesium infusion to try and stabilize the myocardium, our patient continued to show runs of PMVT and required temporary pacing before ICD implantation, the exact role which citalopram played in her presentation to the ED remains unclear.

Citalopram itself was investigated by the Food and Drug Administration (FDA) in the United States in 2011, and this found a dose-dependent increase in QTc prolongation with 20 mg eliciting a mean QTc increase of 8.5 ms [14, 15]. The association with torsades de pointes, however, is not well established, but additional risk factors such as hypokalemia, concomitant QT-prolonging medications, advanced age, and other medical illnesses will be contributing factors in the evolution of this condition [15, 16].

## 4. Conclusion

The presentation of a young fit patient with syncope and seizure-like episodes should always raise concern for the admitting emergency medicine clinician. Prompt review of the ECG and calculation of the QTc should be routinely performed. The patient should be kept on cardiac monitoring and associated risks including potentially harmful medications should be identified promptly. All patients with ventricular ectopy in the setting of cardiac syncope should be discussed with cardiology regarding further investigation.
